# Effect of an Inflatable Air Mattress with Variable Rigidity on Sleep Quality

**DOI:** 10.3390/s20185317

**Published:** 2020-09-17

**Authors:** Hyunsoo Yu, Oh-Soon Shin, Sayup Kim, Cheolsoo Park

**Affiliations:** 1 Department of Computer Engineering, Kwangwoon University, Seoul 01897, Korea; byeng3@kw.ac.kr; 2 School of Electronic Engineering, Soongsil University, Seoul 07040, Korea; osshin@ssu.ac.kr; 3 Human Convergence R&D Department, Korea Institute of Industrial Technology, Ansan 15588, Korea

**Keywords:** sleep, mattress, sleep quality, brain wave, sleep scoring, sleep experiment, rigidity of mattress, mattress design

## Abstract

Several studies, wherein the structure or rigidity of a mattress was varied, have been conducted to improve sleep quality. These studies investigated the effect of variation in the surface characteristics of mattresses on sleep quality. The present study developed a mattress whose rigidity can be varied by controlling the amount of air in its air cells. To investigate the effect of the variable rigidity of the air mattress on sleep quality, participants (Male, Age: 23.9 ± 2.74, BMI: 23.3 ± 1.60) were instructed to sleep on the air mattress under different conditions, and their sleep quality was subjectively and objectively investigated. Subjectively, sleep quality is assessed based on the participants’ evaluations of the depth and length of their sleep. Objectively, sleep is estimated using the sleep stage information obtained by analysing the movements and brain waves of the participants during their sleep. A subjective assessment of the sleep quality demonstrates that the participants’ sleep was worse with the adjustment of the air mattress than that without; however, the objective sleep quality results demonstrates an improvement in the sleep quality when the rigidity of the air mattress is varied based on the participant’s preference. This paper proposes a design for mattresses that can result in more efficient sleep than that provided by traditional mattresses.

## 1. Introduction

Most people spend one-third of their lives sleeping [1]. Furthermore, sleep is essential to maintain good health as it helps revitalize and re-energize the body [2,3,4]. In addition, sleep is associated with weight management and longevity [5,6,7], and sleep quality is associated with self-report health, mood regulation, as well as feelings of anger, confusion, anxiety, and depression [8,9,10,11,12]. In industry, poor sleep quantity leads to high injury rates and loss of productivity [13,14,15]. Consequently, sufficient sleep as well as good sleep quality are critical to ensure good health and overall quality of life [16]. However, Robins et al. reported that solely 19.22% of the adults in the world exhibit good sleep quality [17].

Sleep quality is directly related to health and sleepiness [10]. The key feature of insomnia, which can lead to impairment of immune function [18], cardiac diseases [19], and neurodegenerative disorders [20], is sleep quality [21]. Sleep deficiency results in not only poor health, but also degradation of mental and social functioning [22]. Moreover, poor sleep quality has a negative effect on work productivity and safety [23,24]. Therefore, increasing sleep quality could considerably improve one’s health and quality of life, as well as their productivity at work.

To improve sleep quality, various mattresses have been designed in existing studies [25,26,27]. In a few studies, a thermal controlling device was applied to the developed mattress to improve the sleep quality of the users [28,29,30,31]; others studies have determined the relation between sleep quality and the rigidity of a mattress [27,32,33]. Furthermore, several studies have varied the materials used to design the mattress and have modified its structure [34,35,36]. Some studies investigated users’ preferences on mattresses, where Kim et al. proposed a methodology for finding the appropriate hardness based on various analyzes that measure the users’ experiences with mattresses and found their preferences to different types of mattress comfort were consistent with the outcomes using their methodology [37]. Park et al. investigated the users’ preferences to various mattress shapes corresponding to their postures lying on the mattresses and found that subjective sleeping comfort is related to bed adjustment [38]. In particular, Yoshida et al. reported that there is a relationship between the subjective preference and the stress distribution exerted on the body when a user lies on a mattress [39]. Based on this study, we hypothesized that a mattress customized according to the user’s preference would improve the quality of sleep.

This paper introduces an air mattress whose rigidity can be varied corresponding to the preference of the users. Experiments were conducted to evaluate the effect of the proposed mattress on the quality and quantity of the sleep of its users. These indices are measured based on the users’ subjective sleep assessment and objective sleep scoring index, which are estimated via electroencephalogram (EEG) and accelerometer measurements.

## 2. Methodology

### 2.1. Study Design

**Figure 1:**
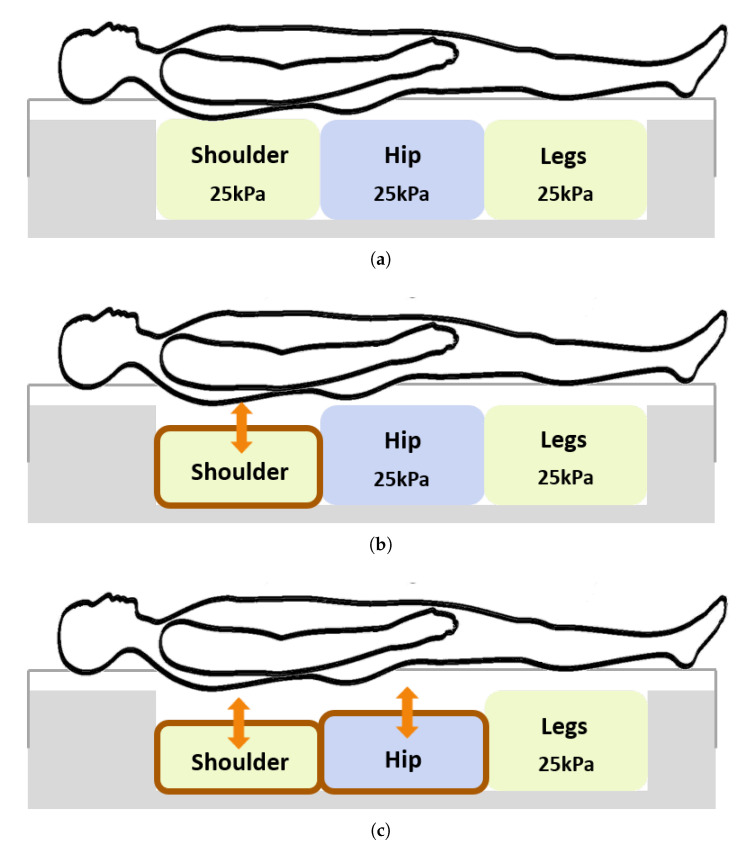
(**a**) Completely filled with pressure condition (A), (**b**) Shoulder customized condition (S); the customized pressure of the air cell at the shoulder was 14.2 ± 4.4 kPa and (**c**) Shoulder—hip customized condition (SH); the customized pressure of the air cell at the shoulder was 15.8 ± 6.4 kPa, and the customized pressure of the air cell at the hip was 15.2 ± 5.5 kPa.

Three conditions were set, as shown in Figure 1, to vary the pressure of the air cells in the mattress. This experiment was conducted to compare sleep quality under three different mattress conditions. Under Condition A, three air cells were completely filled with air and their internal pressures were maintained at 25 kPa. Under Condition S, only the pressure of the air cell at the shoulder was varied corresponding to the user’s preference, and the other two air cells were completely filled. On average, the preferred pressure of the cell at the shoulder was 14.2 ± 4.4 kPa across the participants. Under the SH condition, only the air cell at the legs was completely filled, and the pressures of the other air cells were customized by the participants. The preferred pressures of the cells at the shoulder and hip were 15.8 ± 6.4 kPa and 15.2 ± 5.5 kPa, respectively.

This experiment was approved by the Institutional Review Board of Seoul National University (IRB No. 1906-003-016).

### 2.2. Participants

To score the sleep quality of the participants, EEG and accelerometer data were recorded for 10 male adults who agreed to participate in the experiments. Their ages were 23.9 ± 2.74 and ranges from 21 to 28. Gender was controlled as male following a study that gender could affect sleep quality [40]. The manufactured mattress and air cells were more fit for the body size of the average Korean male, so males were recruited priory in this study. When recruiting participants, they were interviewed to determine whether they met the inclusion and exclusion criteria. The inclusion criterion was that the height is within the range of 170 to 175 cm to fit the size of the mattress. The exclusion criterion was that the participant may be at risk of leaving the mattress due to a bad sleeping habit. Participants with poor sleeping habits were excluded through screening interviews, as Tang et al. did [41]. The average of their BMI scores was 23.3 ± 1.60, and ranges from 20.76 to 25.95. Their Pittsburgh Sleep Quality Index (PSQI) score, which is a self-report questionnaire that assesses sleep quality over a month with a score from 0 to 21, was 5.6 ± 2.22 and the range of their PSQI score was 2 to 10 [42]. Among the participants, there were 5 poor sleepers with a PSQI score higher than 5. To ensure that the participants’ sleep quality was not affected by other factors, the participants were forbidden from consuming caffeine and alcohol for 48 h before the experiment [43,44]. In addition, participants who violate the above policy were excluded from the experiment on the same day through a screening interview before the experiment as Tang et al. did [45]. Second, participants finished their dinners before 7 p.m., had a shower between 9 and 10 p.m., and went to bed at 11 p.m. Lastly, each participant had a gap of 48 h between experiments to ensure that sleep under one mattress condition did not affect sleep under another mattress condition.

### 2.3. Materials

**Figure 2:**
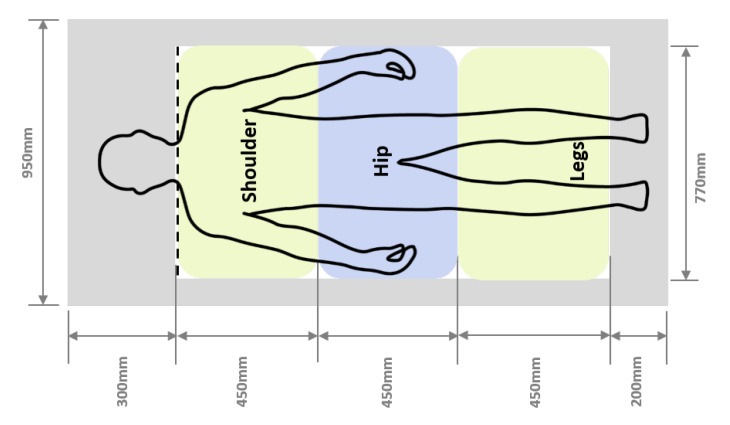
Location and size of air cells in the mattress.

A mattress was developed whose height could be varied by controlling three same-sized air cells in the mattress, as shown in Figure 2. The three air cells are located on the shoulder, hip, and legs of the participants. The size of the air cells and the mattress were designed to fit an average Korean male body [46]. The air cells are covered by latex foam, which is in turn covered by a layer of a bed sheet. The pressures of the air cells are varied using a control box hosed to the air cells, which decides the height of the mattress.

### 2.4. Procedure

**Figure 3:**
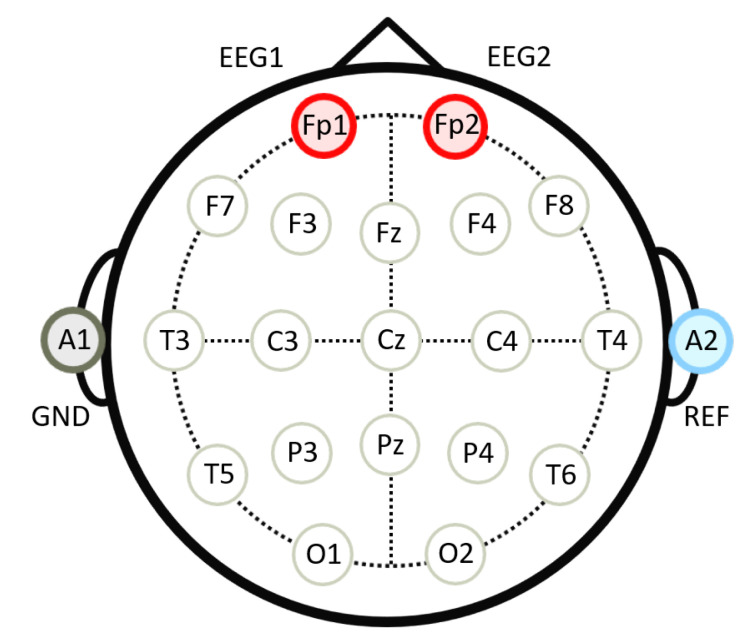
Positions of the attached electrodes.

In this study, sleep scoring was not conducted through polysomnography (PSG), but was conducted through the estimation using a single channel EEG according to [47,48,49]. As shown in Figure 3, four Ag/Ag-Cl electrodes were attached to positions FP1, FP2, A2, and A1 on the participants’ head. The electrodes at positions FP1 and FP2 record the EEG signals; the electrode at position A2 serves as a reference; and the electrode at position A1 is grounded. The two devices used to acquire EEG were BIOPAC MP36 (BIOPAC Systems Inc, Goleta, CA, USA), whose sampling rate was set to 500 Hz, and g.USBamp amplifier (g.tec medical engineering GmbH, Schiedlberg, Austria), whose sampling rate was 600 Hz. In addition, the participants wore wGT3X-BT (ActiGraph LLC, Pensacola, FL, USA) on their wrists such that their movement could be monitored; the sampling rate of the device was 100 Hz.

**Figure 4:**
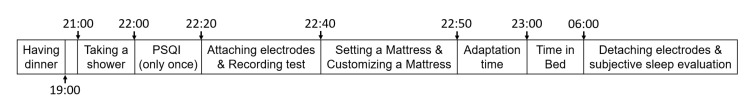
Experimental schedule for a night’s sleep.

To avoid the first night effect [50], all participants slept at the laboratory in the same environment with attaching all electrodes before the actual experiment. The experiment was conducted on three days, and one of three different mattress conditions was considered randomly for each day. Figure 4 shows the procedure of the experiment. On the first day, all the participants answered the PSQI questionnaire before going to bed. Before the electrodes were attached to their respective positions, their exfoliation was removed such that the maximum impedance was 5 kΩ [51]. As shown in Figure 4, the participant had a 10 min adaption time lying on the mattress [52,53]. The participants went to bed at 11 p.m. and slept for 7 h in a room where the temperature and humidity were maintained at 24 ${}^{\circ }$ C and 50% RH, respectively. After waking up at 6 am, the participants responded to a questionnaire designed to help subjectively evaluate their sleep quality. The questionnaire comprised two questions regarding ‘Sleep Length’ and ‘Sleep Depth’, which are the question number 5 and 8 in the “sleep diary” presented by Åkerstedt et al. [54]. For more detailed analysis than the original questionnaire, the selection range was widened from 5 to 7, i.e., a range of 1–7.

### 2.5. Sleep Scoring Algorithm

Subjective sleep assessment was conducted through questionnaires. The length information of each sleep stage was obtained from a hypnogram, as an objective sleep evaluation, where the duration of REM and Deep stages (slow wave sleep, SWS) are the length information of the stages, conventionally used to measure the sleep quality [55]. REM stage maintains the necessary levels of central nervous system activities, promotes a recovery with providing periodic stimulation to the brain [56] and preserves emotional memory sources selectively [57]. SWS induces an endocrine environment that could strongly support the initiation of an adaptive immune response and cleans metabolites [58]. Additionally, RNR (REM to Non-REM ratio) and SSI (Stage Shift index) were calculated as sleep indices corresponding to the sleep quality. Mendonca et al. reported that the higher RNR and the lower SSI result in the improvement of sleep quality [55]. RNR is an index representing the ratio of the duration of the REM stage to that of the non-REM stage, as shown in Equation (1). SSI is an index obtained by dividing the number of times the sleep stage shift during whole sleep by the total sleep time (TST), as shown in Equation (2).

(1) $$RNR\left(\%\right)=\frac{\Sigma \;REM\;(min)}{\Sigma \;nonREM\;(min)}$$

(2) $$SSI=\frac{\Sigma \;Sleep\;Stage\;Shift}{TST\;(h)}$$

To estimate the lengths of the sleep stages, an automatic sleep scoring algorithm is employed, which was introduced in a study by Onton et al. [49,59]. Onton et al. acquired EEG signals from the FP1-A2 and FP2-A2 channels. The signals of one channel were then classified, in terms of 30-s epoch units, into five sleep stages. This was realized by employing the hidden Markov model (HMM) algorithm along with Viterbi and expectation-maximization (EM) algorithms, in an unsupervised manner [60]. The hypnograms of the sleep stages were then estimated. In this study, N1 and N2 were merged to form a Light sleep stage and N3 was considered to be a Deep sleep stage, reducing the number of whole stages from five to four.

A single-channel EEG signal was segmented into 30-s epochs, which served as input to the HMM algorithm [61]; further, the frequency band powers of these epochs were calculated after being filtered using wavelet transform [49,62,63] (Wake: 35–50 Hz, REM: 20–30 Hz, Light: 10.15–15.75 Hz and Deep: 1–3 Hz). There are three parameters for the HMM algorithm, which are an initial probability (π), a transition matrix (*Q*), and an emission matrix (*R*). Since an initial sleep stage always starts with the participant being awake, referring to the paper by Lo et al. [64], the initial probability ($\pi_{0}$) was set as Equation (3) and the initial transition matrix ($Q_{0}$) of the HMM algorithm was initialized as Equation (4). In Equation (4), the element q(i,j) in the matrix represents the probability of transition from the stage *i* to the stage *j* on the next time step. The emission matrix (*R*) comprises the mean (μ) and standard deviation matrices (σ). The mean of the initial emission matrix ($\mu_{0}$) was set as Equation (5). Please note that the probability of one particular frequency band corresponding to a sleep stage (see the diagonal terms in Equation (5)) is higher than those of the others, which was initial expected based on a study by Onton et al. [49]. The standard deviation of the initial emission matrix ($\sigma_{0}$) was set as Equation (6). The parameters (π,Q,R) were updated using the EM algorithm [65], after which the Viterbi algorithm provides estimations of the sleep stages based on the maximum posteriori [66].

(3) $$\pi_{0}=\left[\begin{array}{cccc} 1 & 0 & 0 & 0 \end{array}\right]$$

(4) $$Q_{0}=\left[\begin{array}{cccc} q(W,W) & q(W,R) & q(W,L) & q(W,D)\\ q(R,W) & q(R,R) & q(R,L) & q(R,D)\\ q(L,W) & q(L,R) & q(L,L) & q(L,D)\\ q(D,W) & q(D,R) & q(D,L) & q(D,D) \end{array}\right]=\left[\begin{array}{cccc} 0.75 & 0.01 & 0.24 & 0\\ 0.05 & 0.88 & 0.07 & 0\\ 0.18 & 0.11 & 0.55 & 0.16\\ 0.02 & 0 & 0.14 & 0.84 \end{array}\right]$$

(5) $$\mu_{0}=\left[\begin{array}{cccc} 0.7 & 0.1 & 0.1 & 0.1\\ 0.1 & 0.7 & 0.1 & 0.1\\ 0.1 & 0.1 & 0.7 & 0.1\\ 0.1 & 0.1 & 0.1 & 0.7 \end{array}\right]$$

(6) $$\sigma_{0}=\left[\begin{array}{cccc} 1 & 0 & 0 & 0\\ 0 & 1 & 0 & 0\\ 0 & 0 & 1 & 0\\ 0 & 0 & 0 & 1 \end{array}\right]$$

To validate the HMM algorithm as an automatic sleep scoring method, we obtained a public data set, Sleep-EDF Database Expanded (Sleep-EDFx), containing two EEG channels (Fpz-Cz and Pz-Oz), an electrooculogram (EOG), and an electromyogram (EMG) recorded at a sampling rate of 100 Hz [67,68]. According to the Rechtschaffen and Kales manuals, well-trained technicians manually scored the sleep stages, and thus, the Sleep-EDFx includes all the sleep stages, including wake, REM, S1, S2, S3, and S4. To calculate sleep quality for this data set, S1 and S2 are combined into the light sleep stage, S3 and S4 into Deep stage, reducing the six stages to four: wake, REM, Light, and Deep stages.

The accelerometer data were analyzed using the Sadeh algorithm [69], implemented in ActiLife6 (ActiGraph LLC, Pensacola, FL, USA) software, which estimates sleep and wake states. Sleep efficiency (SE) could be calculated using the formula, SE=Sleep/(Sleep+Wake), based on the estimation of the sleep and wake states. In addition, sleep onset latency (SOL) which is time taken to sleep, wake after sleep onset (WASO) which is total waking time after sleep, and total sleep time (TST) which is total sleep time during time in bed could also be obtained from the actigraphy information [70].

### 2.6. Analyses

To investigate how the proposed mattress affects the quality of sleep, a subjective sleep assessment and objective sleep evaluation were conducted. The subjective sleep assessment was conducted with a questionnaire written about the depth and length of sleep experienced by the participants. To analyze the condition of each mattress, comparative analysis among three different mattress conditions was conducted on the subjective sleep assessment under each mattress condition.

**Figure 5:**
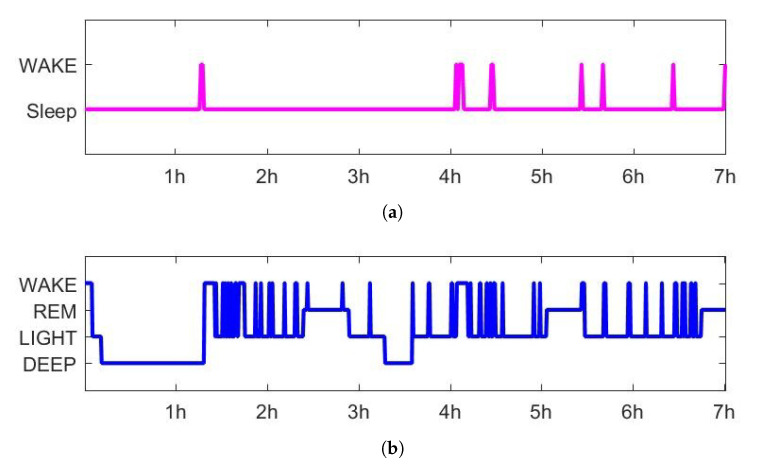
The estimated hypnogram using the (**a**) Sadeh algorithm from the actigraph and (**b**) HMM algorithm from EEG.

Objective sleep evaluation was conducted with various sleep parameters which obtained from actigraphy and EEG analysis. There are two methods for determining the sleep and wake states, which is a binary decision, using actigraphy analysis and EEG analysis as shown in Figure 5a,b. We can get two indices each that can be obtained with wake information such as SE, TST, SOL, and WASO. In this study, comparative analysis is conducted to see how the above indices are affected by the mattress conditions. A hypnogram was estimated using the HMM algorithm, as shown in Figure 5b, and sleep parameters such as the length of REM and Deep, RNR, and SSI were obtained from the estimated hypnogram. A comparative analysis was conducted to investigate these sleep parameters depending on the three mattress conditions.

Since the number of participants in our study is 10, which is less than 30 samples, statistical analysis is performed for validation by nonparametric analysis rather than parametric analysis. Since the results of those sleep parameters in each mattress condition, indicate the conclusion of this study, a nonparametric wilcoxon signed-rank test was conducted between the base condition, A condition, and the condition with the highest score among S and SH condition [71].

There were five poor sleepers with a PSQI score higher than 5, so 10 subjects were divided into 5 poor sleepers and five good sleepers. A comparative analysis of each sleep index is conducted between the two groups to compare and analyze how the customized mattress affects the good sleeper and the poor sleeper.

## 3. Results

### 3.1. Sleep Scoring

**Figure 6:**
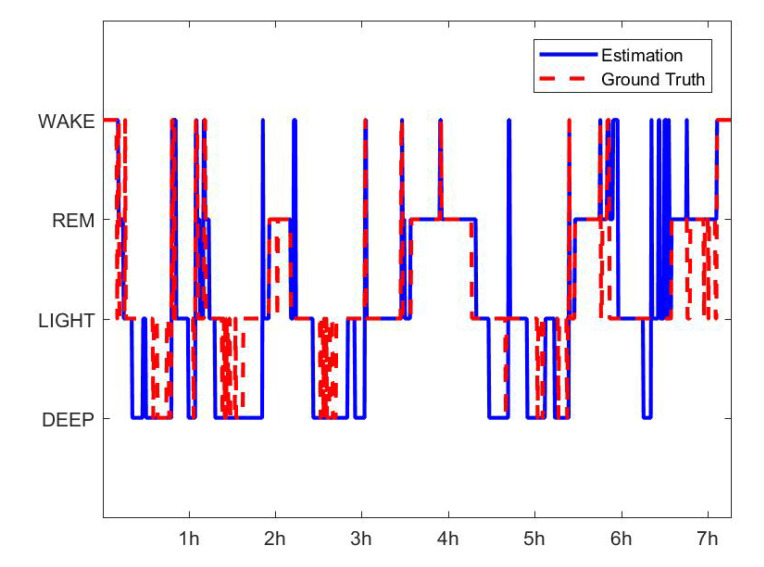
Estimated and true Hypnograms of the Sleep-EDFx public data set (SC4031E0).

Figure 6 is an example of the automatic sleep scoring PSG result for the signal of the Fpz-Cz channel from the public data set, which had 61 participants and a total of 63231 epochs. Using Equation (7), for the Sleep-EDFx data set, the HMM algorithm yielded an accuracy of 89.72%, 86.97%, 77.15%, and 89.58%, respectively, for the four sleep stages of Wake, REM, Light, and Deep, corresponding to the PSG. The accuracy was calculated as a percentage of the number of correct epochs divided by the total number of epochs. In Equation (7), TP, TN, FP, and FN denote true-positive, true-negative, false-positive, and false-negative, respectively [72]. Using these values, precision, recall, and F1 scores were also calculated using Equations (8)–(10), and their results for the Sleep-EDFx data set are summarized in Table 1. The performance of the HMM algorithm in scoring the Wake, REM, and Deep stages exceeded 85%. Various sleep parameters related in wake, REM, Deep were used to measure sleep quality. Light sleep was not considered to measure sleep quality, and thus, this algorithm is suitable for comparing sleep quality under different mattress conditions.

(7) $$Accuracy=\frac{TP+TN}{TP+FP+FN+TN}$$

(8) $$Precision=\frac{TP}{TP+FP}$$

(9) $$Recall=\frac{TP}{TP+FN}$$

(10) $$F1\;score=2\times \frac{Precision\times Recall}{Precision+Recall}$$

### 3.2. Sleep Quality Analysis

**Figure 7:**
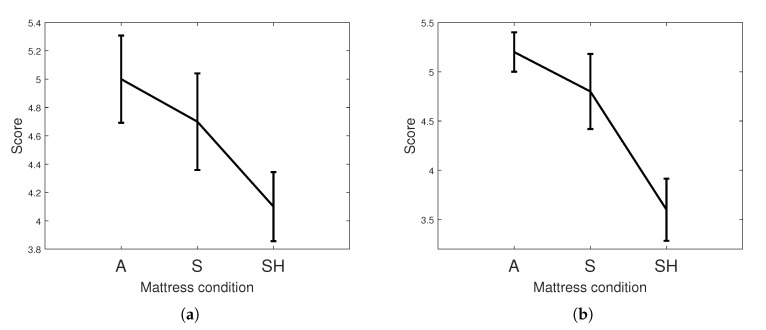
Mean and standard error of the subjective evaluations regarding the (**a**) depth and (**b**) length of sleep on different mattresses under different conditions obtained from the questionnaires to the participants.

After sleeping on different mattresses, the participants qualitatively assessed the length and depth of their sleep. As shown in Figure 7, the participants evaluated that the sleep under Condition A, where all air cells were completely filled with pressure, was the deepest and longest. In contrast, the participants evaluated that their sleep under the SH condition, where the air cells at the shoulder and hip were customized by the participants’ preference, was the shallowest and shortest. In addition, the subjective evaluation of the sleep length and depth under the S condition, where the air cell at the shoulder was customized, was conducted. The value assigned by the participants was between that of the A condition and that of the SH condition. Both of sleep depth and sleep length had statistical significance between A condition and SH condition (*p* < 0.05 for sleep depth, and *p* < 0.01 for sleep length). This finding implies that the participants felt uncomfortable sleeping on the customized mattresses.

As mentioned earlier, the wake and sleep states could be estimated by the participants via the actigraphy and EEG analyses. Using the results obtained, SE, TST, SOL, and WASO [73,74,75] could be calculated to estimate sleep quantity and quality, as summarized in Table 2. These indices contradict the subjective evaluations. Both the actigraphy and EEG analyses yielded the highest TST under the SH condition (389.77 with the actigraphy and 315.75 with EEG) inferring the participants slept in the customized mattress for the longest period. The WASO estimated by both analyses was the shortest under the SH condition (30.2 by the actigraphy and 97.98 by EEG), which indicates that sleep quality was better when the air cells located at the shoulder and hip could be customized. In contrast, the SOL, the period for which the participant is awake before falling asleep, obtained via the EEG analysis indicates that the participants took the longest time to fall asleep under the SH condition; the SOL was 7.4. This might be owing to the difference between their general mattress conditions and the SH mattress condition. Although the actigraphy estimation provides the shortest SOL, this result is not reliable since the sleep stage is determined only based on the movement of the participants irrespective of their actual sleep states.

**Figure 8:**
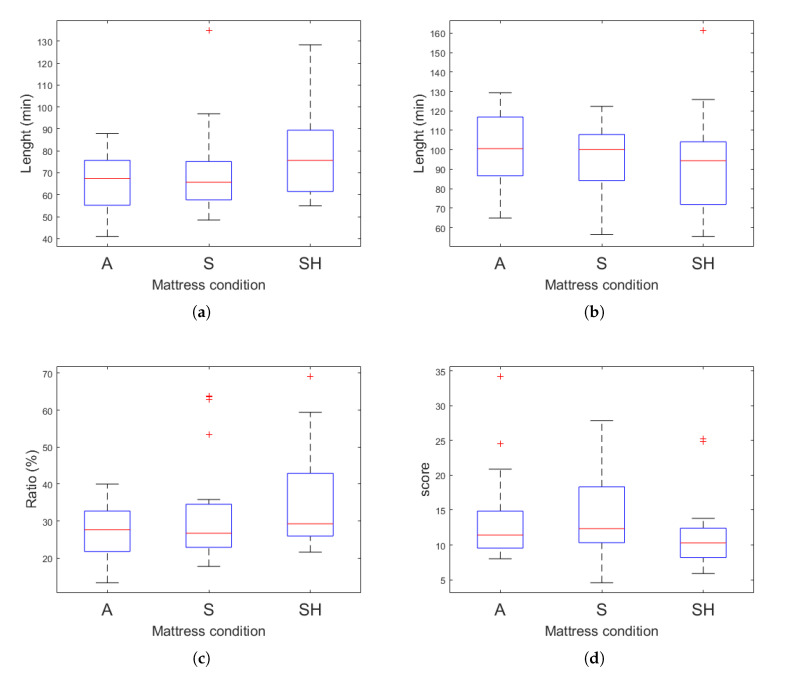
A box plot of the (**a**) duration of REM sleep obtained from the hypnogram estimated via EEG under different mattress conditions, (**b**) duration of Deep sleep obtained from the hypnogram estimated via EEG under different mattress conditions, (**c**) ratio of REM sleep to Non-Rem sleep (RNR) from the hypnogram estimated via EEG under different mattress conditions and (**d**) stage shift index (SSI) obtained from the hypnogram estimated via EEG under different mattress conditions.

Through the EEG analysis, sleep indices from all sleep stages (Wake, REM, Light and Deep) could be estimated. Mendonca et al. reported that the higher the lengths of REM sleep, and Deep stages, and RNR were, the higher sleep quality was, while the lower SSI yielded the better one [55]. In our experiment, REM period under the customized SH condition was significantly longer than that under the uncustomized A condition, as shown in Figure 8a, which was tested using wilcoxon signed-rank test (*p* < 0.05). Although the length of Deep stage in the A condition was slightly longer than that in SH condition, there was no statistical significance as shown in Figure 8b. The results of the comparative analysis of RNR were similar to the trend of those from the REM stage analysis. Significantly higher RNR in the SH condition was found compared with that in the A condition as shown in Figure 8c (*p* < 0.05). Additionally, Figure 8d displays SSI was lower in the SH condition meaning that the sleep quality was improved compared with the A condition.

The participants we recruited consisted of good and poor sleepers. Since the factors of the above comparative analysis such as objective sleep and subjective sleep assessment may be affected by the participants’ usual sleep quality, a comparative analysis was conducted divided into two groups. As a result, both the good sleeper and the poor sleeper evaluated similar trends for those three mattress conditions as shown in Table 3. On the other hand, based on the objective sleep evaluation, the results of REM and RNR of the poor sleepers were higher in the SH condition higher compared with the A condition, which are tested using the nonparametric wilcoxon signed-rank test (*p* < 0.01 for the length of REM stage and *p* < 0.05 for RNR) as shown in Table 3. Similarly, the poor sleepers had lower SSI in the SH condition than that in the A condition, inferring the improvement of the sleep quality, which was statistically significant with *p*-value less than 0.01. On the other hand, the Deep stage parameters of the poor sleepers had the higher mean value in the A condition, but it was not significant compared with those of the other conditions. These results demonstrates that the mattress we proposed helps improve the sleep quality in terms of REM, RNR and SSI parameters, particularly for the poor sleepers.

## 4. Discussion

The objective sleep evaluation in Table 3 illustrated significant improvement of sleep quality for the poor sleepers using the customized mattress was more effective in improving the sleep quality. However, the good sleepers were not affected by the different conditions of the mattress. This might be able to their high quality of sleep, which could not have a chance to be improved any further. In addition, it should be noted that the REM, RNR, and SSI indices in poor sleeper were higher than those of good sleepers in SH condition, and SSI particularly showed statistical significance. (*p* < 0.05). This suggests that even a poor sleeper could improve the sleep quality more than a good sleeper using the customized sleep environment.

The subjective evaluation demonstrates that the more the mattress was customized, the lower the sleep quality. However, the objective measure of SE obtained for the completely customized mattresses yielded the highest value via the actigraphy and EEG analyses. When using Spearman’s Roh, which calculates the correlation coefficient between the two parameters in a nonparametric method, a relationship between objective sleep indices and subjective sleep assessment could not be found, as shown in Table 4. This result indicates that the length and sleep quality assumed by the participants could differ from the practical experience they had. Tonetti et al. conducted an objective sleep evaluation using actigraphic parameters and subjective sleep evaluation using MSQ questionnaire on spring mattresses and their proposed latex mattresses [34]. The study showed a discrepancy between the subjective and objective sleep evaluations. Bader et al. conducted an objective sleep evaluation using sleep parameters obtained from BCG signals and a subjective sleep evaluation obtained from self-made questionnaires on soft and hard mattresses [32]. In that study, there was also a discrepancy between the subjective and the objective sleep evaluation. They suggested that there might be little relationship between the quality of sleep experienced by the participants and the quality of sleep measured by the existing sleep parameters.

This study has several limitations. Their average PSQI is 5.6, which is a higher score than the cut-off value of PSQI. In other words, the participants were biased by poor sleeper. Also, only 10 participants were recruited. Since the number of participants was 10, when the participants was divided into two groups, poor sleeper and good sleeper, there were only 5 people in one group. More meaningful results could be obtained if more participants are recruited in future study.

Future studies should focus on automating the process to customize the mattress. In this study, customization was realized based on the feedback of the participants; the experimenters were adjusting the pressure of the air cells. The control of the pressure in the air cells can be completely automated, corresponding to the comforts of the users, using additional sensors such as pressure of their bodies on the mattress. Maximum sleep quality can then be provided to the participants. In addition, the shape of the mattress needs to be customized in real time. In this study, the air cells were customized and fixed while the participants were in a supine position, which might discomfort the participants when they changed their sleep positions. If the shape of the mattress is customized in real time while the participants slept, their sleep quality could increase.

## 5. Conclusions

A mattress whole shape that could be customized was proposed in this paper. Furthermore, its performance for improving sleep quality was investigated. The quantitative results obtained demonstrated that objective users’ sleep quality on the mattress customized to their preference was higher than that on the uncustomized mattress. In other words, varying the shape of a mattress based on users’ preferences affected their sleep quality.

## Figures and Tables

**Table 1:** Accuracy, precision, and recall for all four sleep stages, yielded by the HMM algorithm for the Sleep-EDFx public data set with 61 participants consisting of 63,231 epochs.

	Wake (%)	REM (%)	Light (%)	Deep (%)
**Accuracy**	87.72	86.97	77.15	89.58
**Precision**	65.18	61.16	92.02	58.59
**Recall**	80.52	83.46	60.27	87.19
**F1 score**	72.05	70.63	72.85	72.09

**Table 2:** Sleep efficiency (SE), total sleep time (TST), sleep onset latency (SOL), and wake after sleep onset (WASO) via the actigraphy signal analysis and the EEG signal analysis.

		Mean (SD)		
		A	S	SH
**Actigraphy**	**SE (%)**	91.08 (8.82)	91.32 (5.81)	92.80 (6.01)
	**TST (min)**	382.57 (37.04)	383.56 (24.40)	389.77 (25.25)
	**SOL (min)**	1.1 (0.31)	1.2 (0.62)	1.0 (0)
	**WASO (min)**	36.1 (35.64)	35.6 (24.29)	30.2 (25.33)
**EEG**	**SE (%)**	74.87 (8.08)	72.54 (9.30)	75.18 (8.30)
	**TST (min)**	314.45 (33.95)	304.66 (39.04)	315.75 (34.86)
	SOL (min) *	4.05 (4.28)	7.33 (13.02)	7.4 (6.32)
	**WASO (min)**	102.63 (34.67)	109.15 (31.53)	97.98 (34.37)
**Questionnaire**	Depth *	5.0 (1.38)	4.7 (1.53)	4.1 (1.25)
	Length **	5.2 (0.89)	4.8 (1.70)	3.6 (1.47)

Significance codes: * p<0.05, ** p<0.01. Significance between A condition and SH condition.

**Table 3:** Comparison of subjective and objective sleep evaluation between poor sleepers and good sleepers.

			Mean (SD)		
			A	S	SH
**Poor sleeper**	**Subjective**	**Depth**	5.4(1.26)	5.2(1.23)	4.2(1.23)
		Length *	5.6 (5.16)	5.4 (1.26)	4.6 (1.08)
	**Objective**	REM **	58.03 (12.03)	66.82 (16.94)	81.05 (20.39)
		**Deep**	101.08 (17.38)	85.00 (19.01)	84.95 (31.58)
		RNR *	23.87 (8.65)	34.66 (18.32)	36.65 (14.20)
		SSI **	15.23 (8.03)	14.64 (8.01)	8.80 (1.75)
**Good sleeper**	**Subjective**	**Depth**	4.6 (1.43)	4.2 (1.69)	4.0 (1.33)
		Length **	4.8 (1.03)	4.2 (1.93)	2.6 (1.08)
	**Objective**	**REM**	73.51 (13.13)	74.51 (22.69)	76.81 (20.14)
		**Deep**	101.68 (19.52)	105.42 (12.50)	100.03 (17.67)
		**RNR**	30.79 (6.19)	30.58 (12.31)	33.09 (13.21)
		**SSI**	12.34 (4.90)	13.71 (4.17)	14.03 (6.12)

Significance codes: * p<0.05, ** p<0.01. Significance between A condition and SH condition.

**Table 4:** The correlation coefficient using Spearman’s Roh between subjective sleep assessment (Depth, Length) and objective sleep evaluation (SE, TST, SOL, WASO, REM, Deep, RNR, and SSI).

	ACT-SE	ACT-TST	ACT-SOL	ACT-WASO	REM	Deep
**Depth**	−0.2400	−0.2400	−0.0674	0.2269	0.0781	−0.0695
**Length**	−0.1532	−0.1532	0.0024	0.1382	−0.1429	0.1398
	**EEG-SE**	**EEG-TST**	**EEG-SOL**	**EEG-WASO**	**RNR**	**SSI**
**Depth**	0.2733	0.0163	−0.0717	−0.0137	0.0375	0.0773
**Length**	0.0419	0.0419	−0.2076	0.0084	−0.1700	−0.1204

## Abbreviations

The following abbreviations are used in this manuscript:

BMI	Body mass index
EEG	electroencephalogram
PSQI	Pittsburgh Sleep Quality Index
PSG	Polysomnography
FP(1 or 2)	Frontal-pole on the location of scalp electrodes
A(1 or 2)	Auricular point on the location of scalp electrodes
SWS	Slow wave sleep
RNR	REM to Non-REM ratio
SSI	Stage shift index
SE	Sleep efficiency
TST	Total sleep time
SOL	Sleep onset latency
WASO	Wake after sleep onset
EM algorithm	expectation-maximization algorithm
HMM	Hidden Markov model
EOG	Electrooculogram
EMG	Electromyogram
REM sleep	Rapid eye movement sleep
TP	True-positive
TN	True-negative
FP	False-positive
FN	False-negative
BCG	Ballistocardiogram
MSQ	Mini sleep questionnaire
